# Supplementary figures and images for: Instance-level quantitative saliency in multiple sclerosis lesion segmentation
Source: Sci Rep. 2026 Feb 2;16:6917. doi: 10.1038/s41598-026-36560-9 (PMC12916819; doi:10.1038/s41598-026-36560-9)

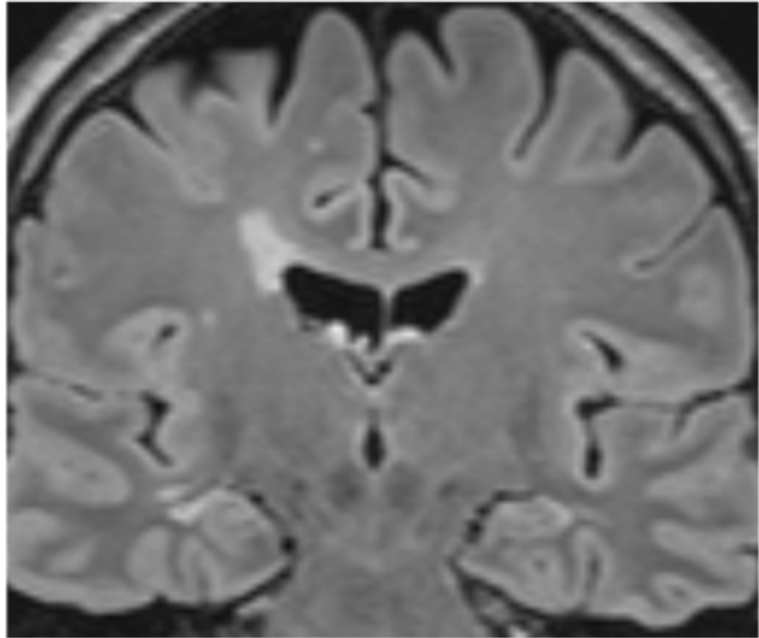

(a)

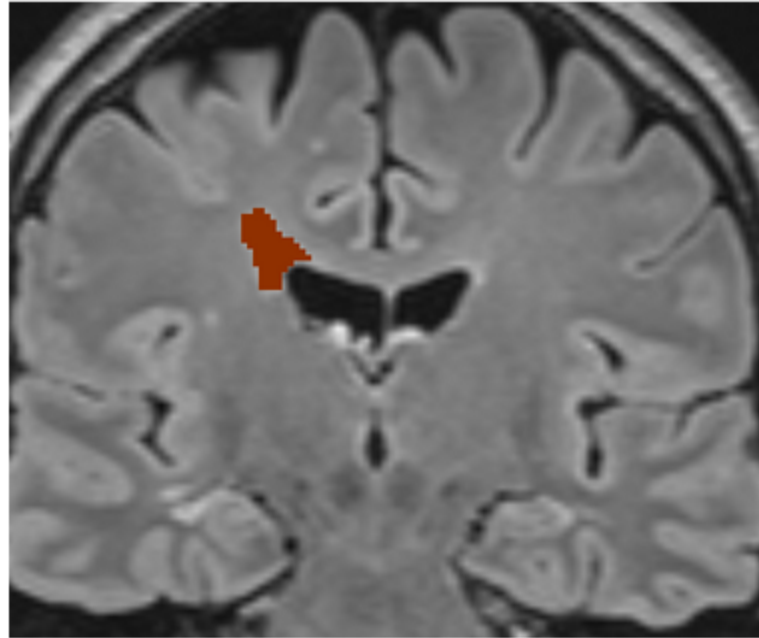

(b)

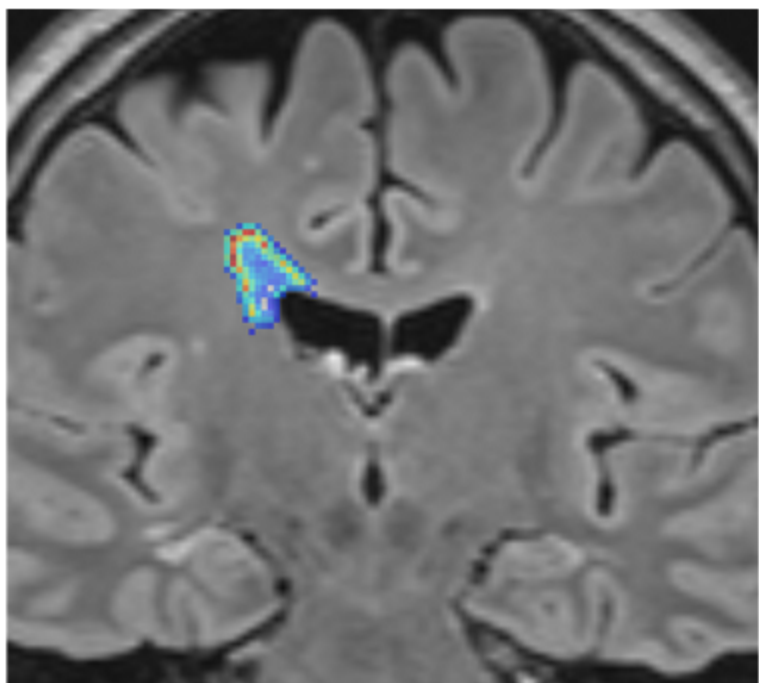

(c)

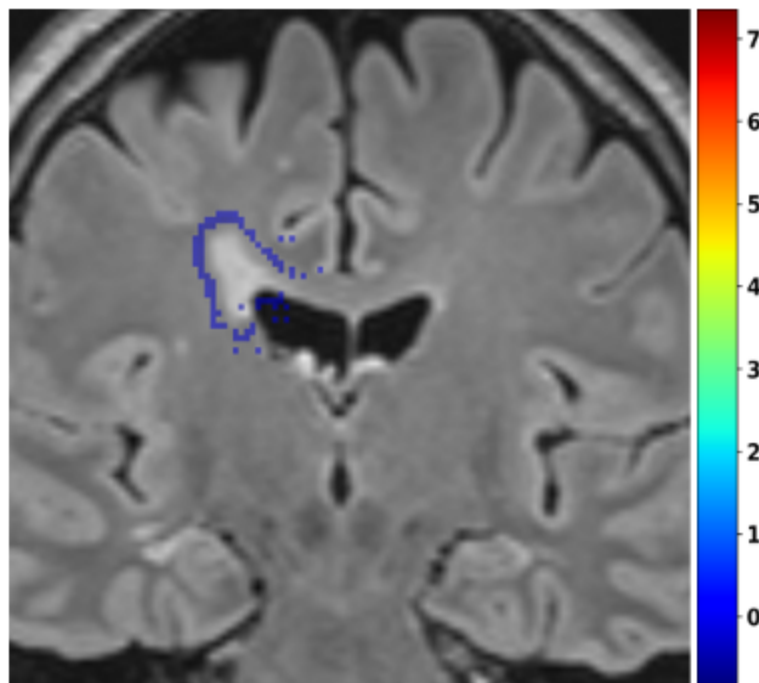

(d)

Supplement: Supplementary file 1 — Supplementary Information 1. [file 41598_2026_36560_MOESM1_ESM.pdf]

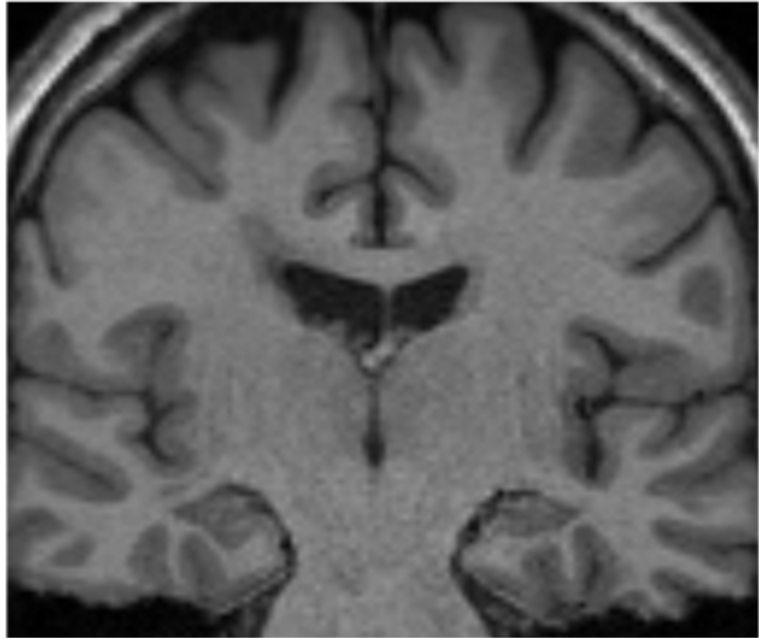

(a)

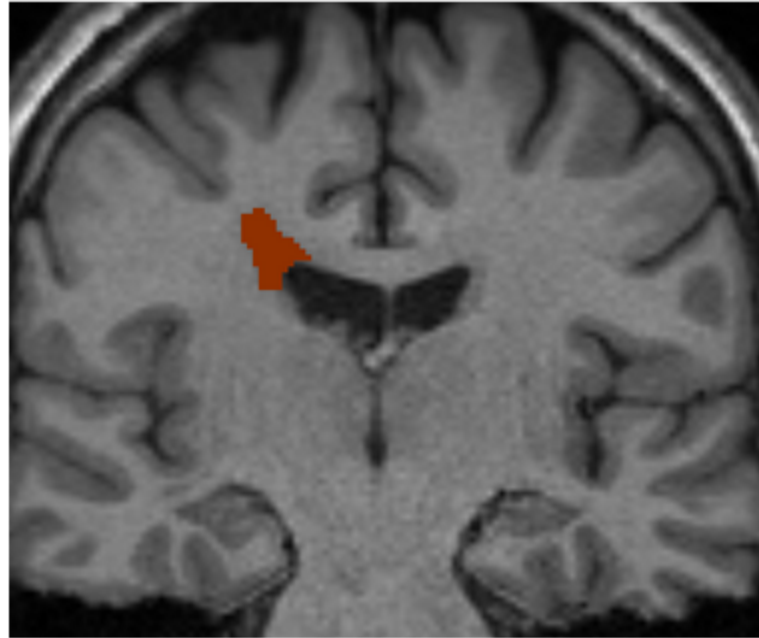

(b)

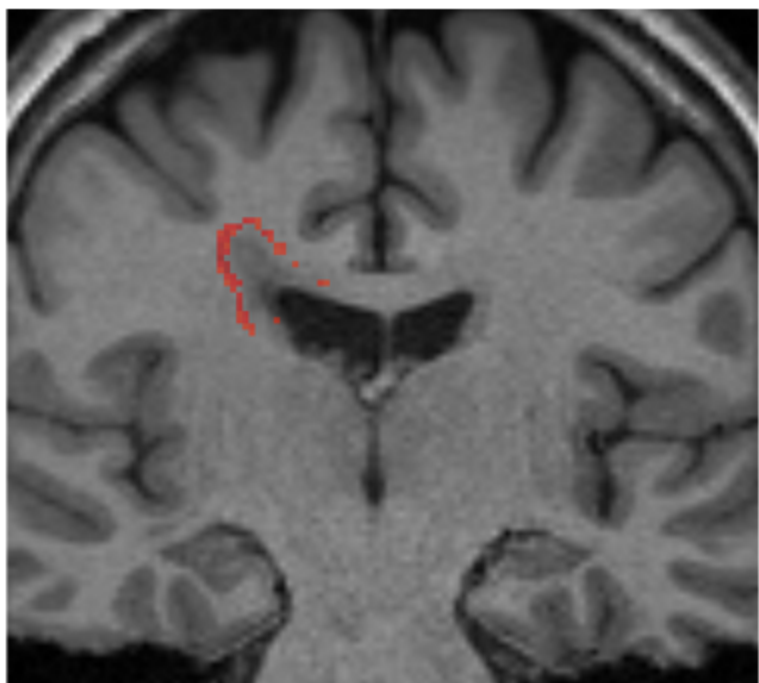

(c)

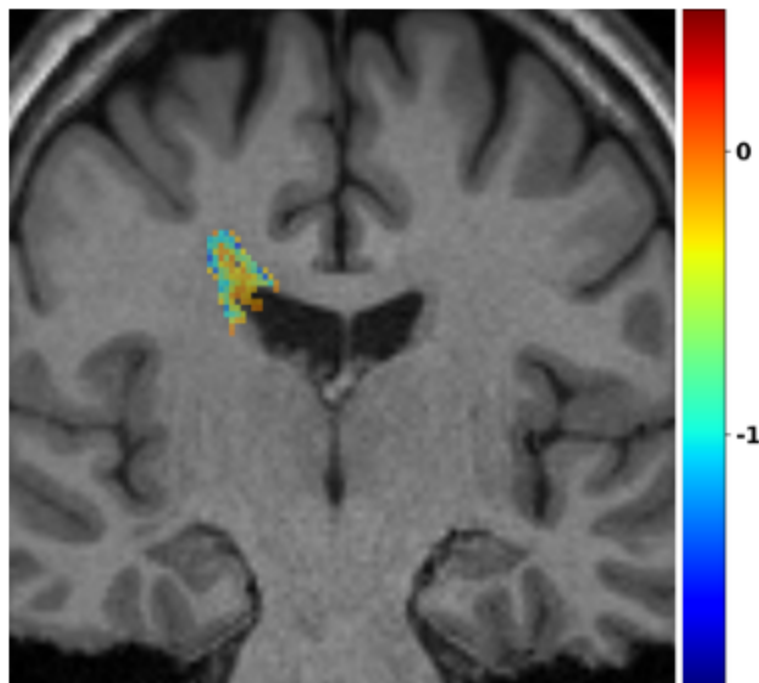

(d)

Supplement: Supplementary file 2 — Supplementary Information 2. [file 41598_2026_36560_MOESM2_ESM.pdf]

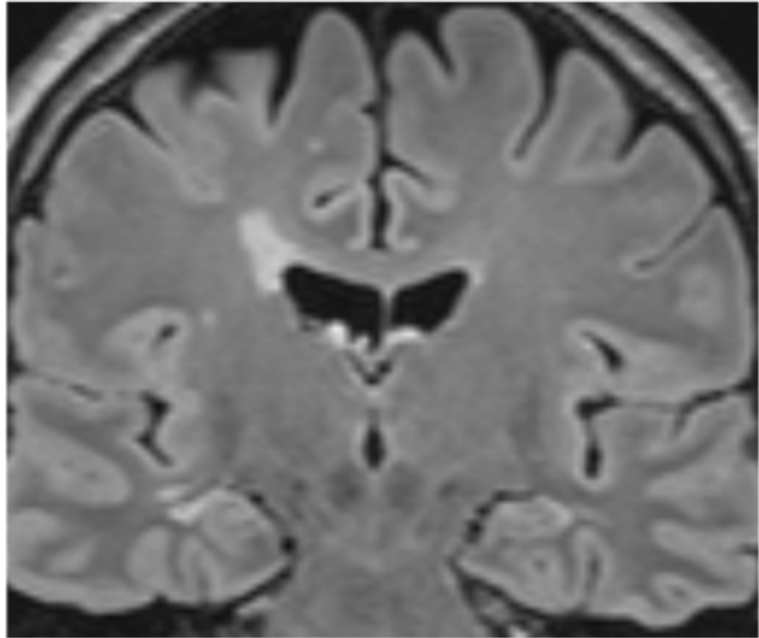

(a)

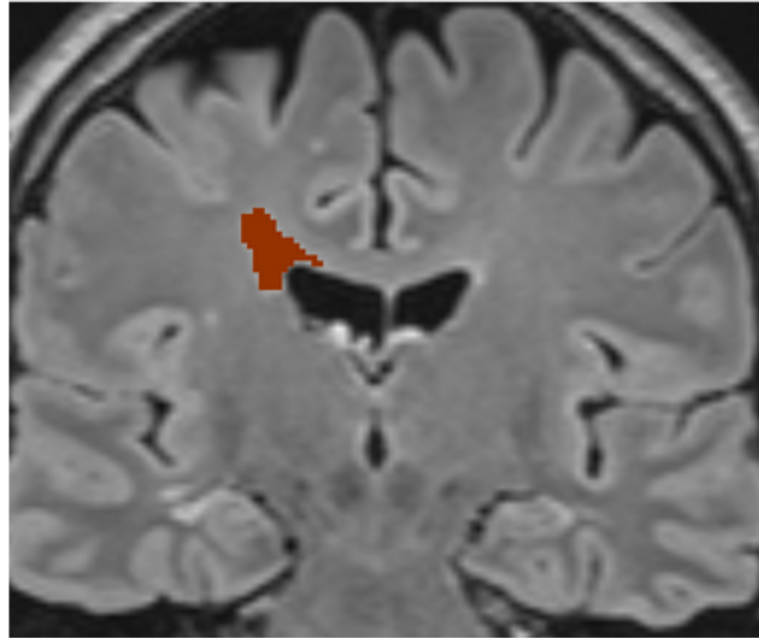

(b)

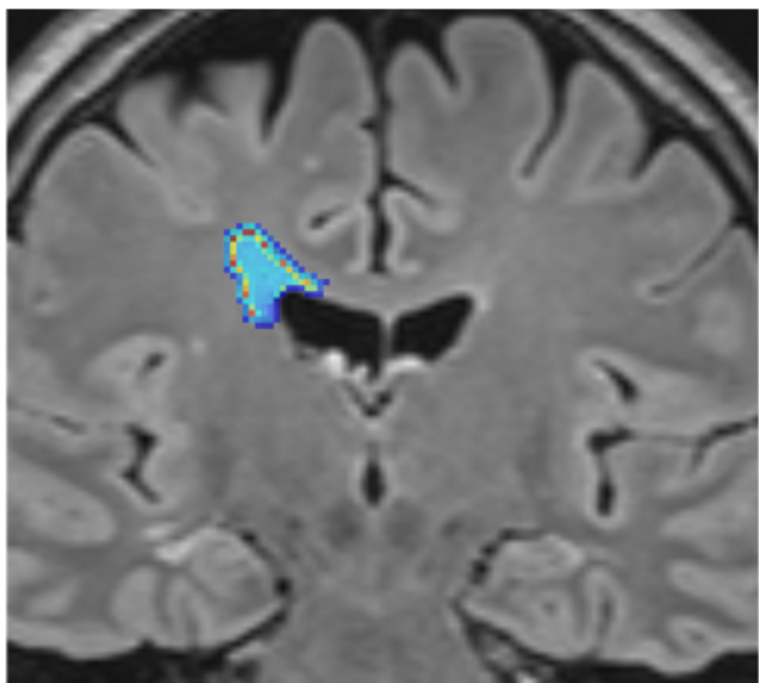

(c)

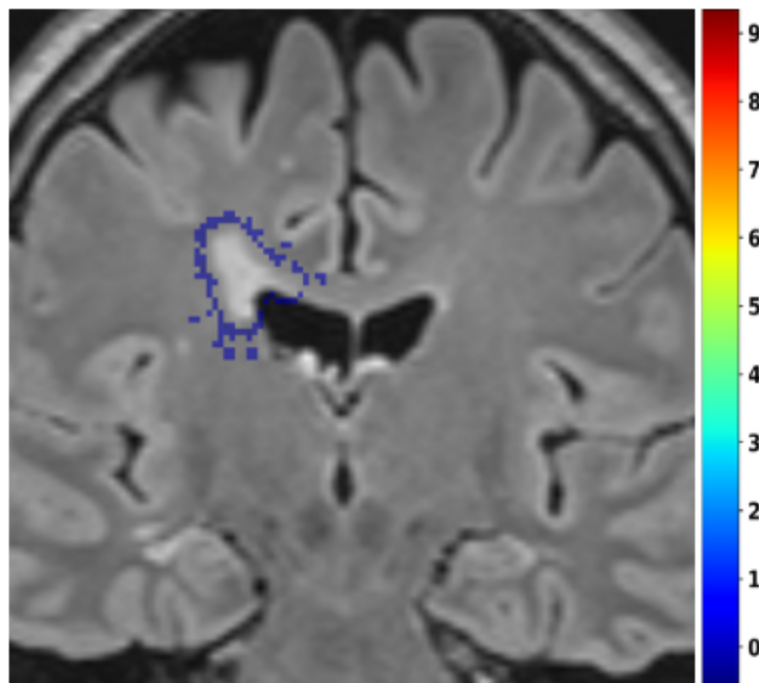

(d)

Supplement: Supplementary file 3 — Supplementary Information 3. [file 41598_2026_36560_MOESM3_ESM.pdf]

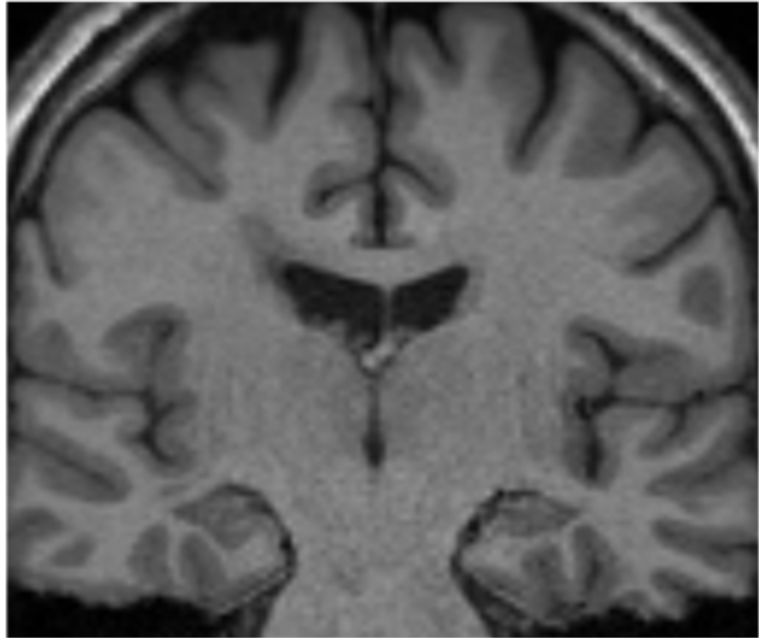

(a)

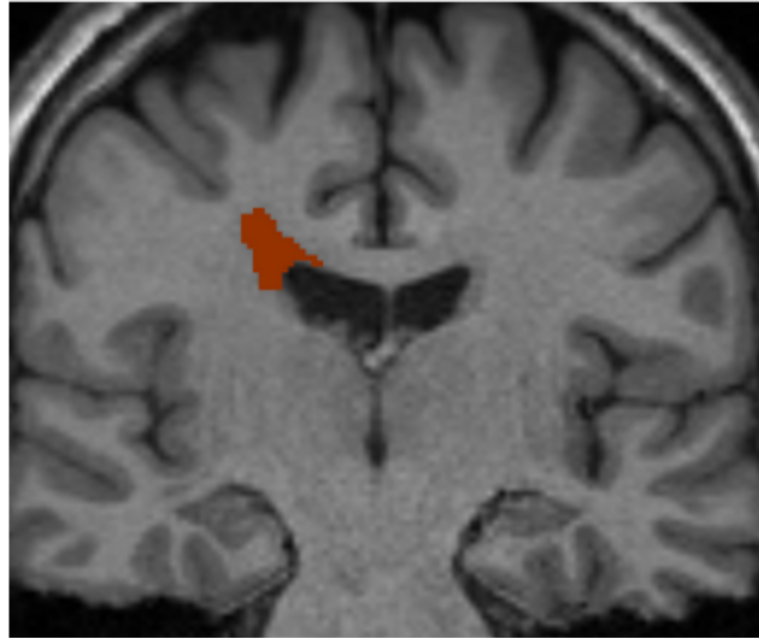

(b)

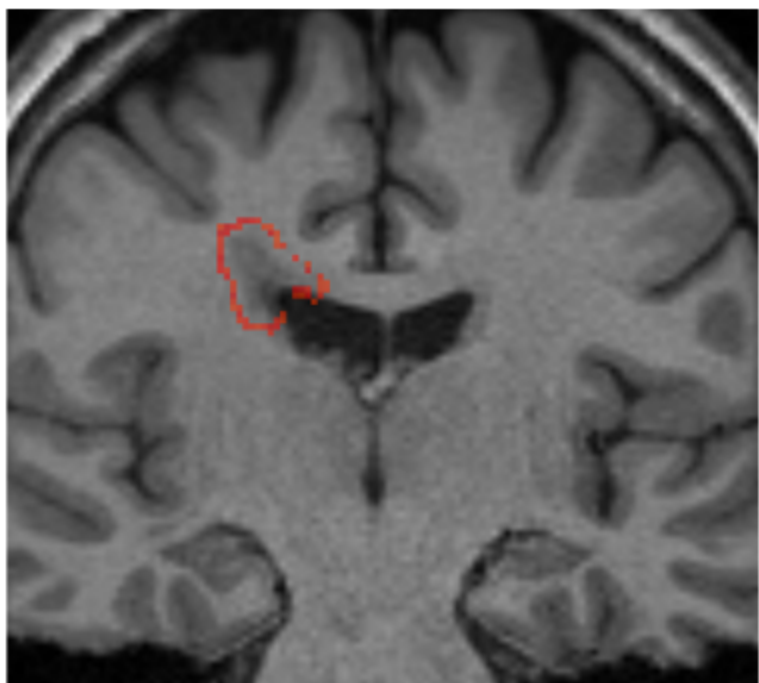

(c)

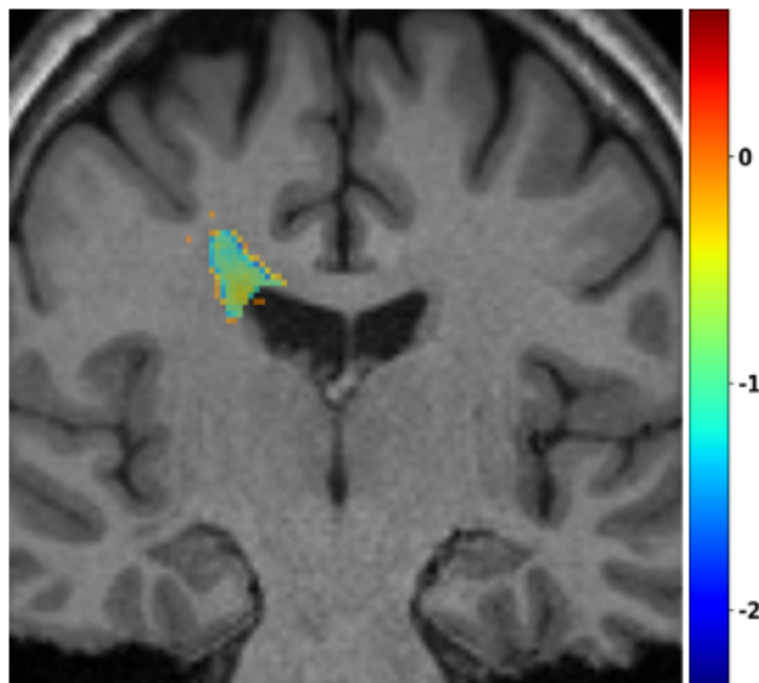

(d)

Supplement: Supplementary file 4 — Supplementary Information 4. [file 41598_2026_36560_MOESM4_ESM.pdf]
